# Binding of *eEF1A2* to the RNA-dependent protein kinase PKR modulates its activity and promotes tumour cell survival

**DOI:** 10.1038/s41416-018-0336-y

**Published:** 2018-11-13

**Authors:** Alejandro Losada, María José Muñoz-Alonso, Marta Martínez-Díez, Federico Gago, Juan Manuel Domínguez, Juan Fernando Martínez-Leal, Carlos M. Galmarini

**Affiliations:** 1 Departamento de Biología Celular y Farmacogenómica, Pharma Mar S.A., Colmenar Viejo, 28770 Madrid, Spain; 2 Departamento de Ciencias Biomédicas, Unidad Asociada al IQM-CSIC, Universidad de Alcalá, Madrid, Spain

**Keywords:** Oncogenes, Cell signalling

## Abstract

**Background:**

Through several not-fully-characterised moonlighting functions, translation elongation factor *eEF1A2* is known to provide a fitness boost to cancer cells. Furthermore, *eEF1A2* has been demonstrated to confer neoplastic characteristics on preneoplastic, nontumourigenic precursor cells. We have previously shown that *eEF1A2* is the target of plitidepsin, a marine drug currently in development for cancer treatment. Herein, we characterised a new signalling pathway through which *eEF1A2* promotes tumour cell survival.

**Methods:**

Previously unknown binding partners of *eEF1A2* were identified through co-immunoprecipitation, high-performance liquid chromatography-mass spectrometry and proximity ligation assay. Using plitidepsin to release *eEF1A2* from those protein complexes, their effects on cancer cell survival were analysed in vitro.

**Results:**

We uncovered that double-stranded RNA-activated protein kinase (PKR) is a novel *eEF1A2*-interacting partner whose pro-apoptotic effect is hindered by the translation factor, most likely through sequestration and inhibition of its kinase activity. Targeting *eEF1A2* with plitidepsin releases PKR from the complex, facilitating its activation and triggering a mitogen-activated protein kinase signalling cascade together with a nuclear factor-κB-dependent activation of the extrinsic apoptotic pathway, which lead to tumour cell death.

**Conclusions:**

Through its binding to PKR, *eEF1A2* provides a survival boost to cancer cells, constituting an Achilles heel that can be exploited in anticancer therapy.

## Introduction

Human translation elongation factor 1α2, encoded by the *eEF1A2* gene, has been identified as a pro-oncogenic protein. Absent from the majority of body tissues, with the exceptions of brain, heart and skeletal muscle^1^ (tissues with very low cell death), it is expressed in many cancer types,^1–3^ where it provides tumour cells with improved fitness and survival. Moreover, *eEF1A2* has been demonstrated to confer neoplastic characteristics to preneoplastic, nontumourigenic human ovarian precursor cells^4^ and to NIH-3T3 mouse fibroblasts.^2^ Although the canonical function of *eEF1A2* is delivering aminoacyl-transfer RNAs to the ribosome during translation, other “moonlighting” functions have been described for the protein.^5^ For example, sphingosine kinase 1 (SPHK1) activity is enhanced by direct interaction with the guanosine diphosphate (GDP)-bound form of *eEF1A2*^6,7^ favouring tumour cell growth. Similarly, *eEF1A2* enhances peroxiredoxin-1 (PRDX1) activity, providing cells with extraordinary resistance to oxidative stress-induced cell death.^8^ We have recently reported that *eEF1A2* is the molecular target for plitidepsin, a marine drug under development for the treatment of cancer patients.^9^ We have shown that plitidepsin binds with high affinity (*K*_D_ ~80 nM) and persistence (residence time ∼9 min) to *eEF1A2*.^9^ While a contribution to oncogenicity of *eEF1A2*’s canonical function cannot be ruled out, it seems clear that some other functions of the protein are essential to convey its pro-tumourigenic effect.^8,10–12^

Here we investigate the role of new “moonlighting functions” of *eEF1A2* in the maintenance of tumour phenotype. Using plitidepsin to target *eEF1A2*, we demonstrate that the pro-oncogenic activities of *eEF1A2* are important for the fitness and survival of tumour cells. Most unexpectedly, a previously undescribed regulatory interaction between *eEF1A2* and interferon-induced, dsRNA-activated protein kinase (PKR, EIF2AK2) has been found to be disrupted by the binding of plitidepsin to the elongation factor. This new *eEF1A2*–PKR complex has been proven essential for the survival of cancer cells. In addition, binding of plitidepsin to *eEF1A2* inhibits its interaction with PRDX1 and blocks the activation of SPHK. Taken together, these results show that the fitness boost that the moonlighting functions of *eEF1A2* provide to cancer cells constitutes an Achilles heel that can be purposely exploited in anticancer therapy.

## Materials and methods

### Reagents

Plitidepsin (CAS No. 137219-37-5) was synthesised at PharmaMar (Madrid, Spain). Poly(I:C) was from InvivoGen (San Diego, CA, USA). C16, BAY 11-7082, PF-543, and anti-FAS (CH11) antibodies (Abs) were from Merck-Millipore (Danvers, MA, USA). PRDX1-Myc plasmid, anti-myc-tag and anti-tGFP-tag magnetic beads and anti-tGFP monoclonal Abs were from Origene (Rockville, MD, USA). hTNF-α (human tumour necrosis factor-α), anti-phospho-JNK (c-Jun N-terminal kinase), anti-phospho-p38, anti-myc-tag, anti-phospho-eIF2α, anti-eIF2α, anti-NF-κB p65, anti-IκBα, anti-BCL2, anti-c-Myc, anti-cyclin-D1, anti-FAS, anti-Mcl1, anti-caspase-8, and anti-FLAG Abs were from Cell Signaling Technologies (Danvers, MA, USA). Anti-*eEF1A2* Ab was from GeneTex (Irvine, CA, USA). Anti-FAS (SM1/23) Ab was from Enzo Life Sciences (Farmingdale, NY, USA). Anti-XIAP Ab was from BD (San Jose, CA, USA). Step Human High-Yield IVT, anti-poly(ADP-ribose) polymerase (PARP) Ab, Alexa-Fluor^®^-488 goat anti-rabbit IgG, pT7CFE1-NHis-GST-CHA vector, glutathione agarose and glutathione *S*-transferase (GST)-tagged human rhinovirus-3C protease were from Thermo Scientific (Rockford, IL, USA). Anti-SPHK1, anti-SPHK2, anti-PKR and anti-phospho-PKR Abs were from Abcam (Cambridge, UK). Anti-*eEF1A2* Ab for proximity ligation assay (PLA) was purchased from Santa Cruz Biotechnology (Dallas, TX, USA). Lipofectamine^®^ 2000 and Opti-MEM^™^ were from Life Technologies (Carlsbad, CA, USA). Bright-Glo™ and ADP-Glo™ were from Promega (Madison, WI, USA). SPHK Activity Assay and Sphingosine-1-phosphate (S1P) Competitive ELISA Kits were from Echelon Biosciences (Salt Lake City, UT, USA). All other reagents, including Duolink^®^ PLA, were purchased from Sigma-Aldrich (St Louis, MO, USA).

### Cell culture

HeLa cervix adenocarcinoma (ATCC CCL-2) cells were purchased from ATCC (Manassas, VA, USA). Cell lines were further authenticated through the AACR authentication service. Plitidepsin-resistant HeLa cells (HeLa-APL-R) and stably transfected HeLa APL-A2-tGFP and APL-A1-tGFP cells were generated at PharmaMar.^9,13^ PKR-null (PKR^−/−^) and eIF2α S51A mutant mouse embryonic fibroblasts (MEFs) and their wild-type (wt) counterparts were a gift from César de Haro (CBMSO, Madrid, Spain). Cell culture, proliferation assays, transfection and clone selection procedures were described elsewhere.^8^

### Immunoprecipitation

Cells were lysed with lysis buffer (1% Triton X-100, 50 mM Tris-HCl, pH 7.4, 150 mM NaCl, 1 mM EDTA) and centrifuged at 13,000 × *g* for 20 min. Supernatant protein was quantified and 150 to 500 µg of total protein was immunoprecipitated overnight at 4 °C with appropriate Ab-coated beads. Beads were extensively washed, boiled for 10 min in Laemmli buffer, subjected to polyacrylamide gel electrophoresis, electro-blotted onto polyvinylidene fluoride membranes and hybridised with the appropriate antibodies. Immunoreactive bands were analysed with a Bio-Rad ChemiDoc Touch Imager (Hercules, CA, USA). Densitometry for the quantification of the immunoblotting bands was performed with the Bio-Rad Image Lab v.6 software (Hercules, CA, USA). Experiments were repeated at least three times to ensure consistency.

### Immunofluorescence

Cells (25,000 per well) were cultured in 16-well slides and treated with vehicle, 450 nM plitidepsin or 25 ng/mL hTNF-α for the indicated times. Then, cells were washed with phosphate-buffered saline (PBS), fixed for 10 min with 4% paraformaldehyde, washed with PBS and permeabilised for 10 min with PBS-0.2% Triton X-100 at room temperature (RT). Slides were blocked with 5% bovine serum albumin (BSA) at RT for 1 h and incubated with anti-NF-κB Ab (1:100 in 5% BSA–0.2% Triton X-100) for 1 h at RT. Slides were then washed with PBS–0.2% Triton X-100 and incubated for 1 h with Alexa Fluor^®^ 488 goat anti-rabbit IgG diluted 1:200 in 5% BSA–0.2% Triton X-100. Slides were finally rinsed with PBS–0.2% Triton X-100, mounted and observed through a Zeiss AxioVert 200M Apotome microscope (Oberkochen, Germany). Images were acquired using the AxioVision Release 4.8.2.SP2 software.

### Proximity ligation assay

For the assay, Duolink^®^ PLA reagents and protocol were used following the instructions of the supplier. Sixteen-well slides were seeded with 40,000 HeLa wt cells and cultured for 24 h before being treated either with vehicle (0.1% dimethyl sulfoxide) or 450 nM plitidepsin for 1 h. Slides were then stained with the appropriate antibodies following the instruction of the manufacturer, mounted and observed through a Zeiss AxioVert 200M Apotome microscope (Oberkochen, Germany). Images were acquired using the AxioVision Release 4.8.2.SP2 software.

### PKR activity assay

*eEF1A2* was purified from rabbit muscle as described in ref.^9^; rabbit and human *eEF1A2* sequences are 100% identical. *eEF1A2* from the muscle was mainly in GDP-bound conformation. To determine GDP and guanosine triphosphate (GTP) occupation within *eEF1A2* and to obtain the GTP-bound conformation by exchanging GDP with the non-hydrolyzable GTP analogue GppNHp, we followed the procedures described by Smith and Rittinger.^14^

Human PKR (HGNC:9437) was cloned into pT7CFE1-NHis-GST-CHA vector. PKR was produced using the Step Human High-Yield IVT Kit. The identity of the protein was confirmed by Western blot. Typically 1 mL of translation reaction using 40 µg of plasmid yielded 100 µg of recombinant protein.

PKR activity was monitored using myelin basic protein (MBP) as substrate, measuring the adenosine diphosphate (ADP) produced after 60 min. Reactions were performed in a final volume of 5 µL with 80 nM PKR, 10 µM ATP and 5 µM MBP in 25 mM Tris-HCl, pH 7.5, 20 mM MgCl_2_, 500 µM dithiothreitol and in the presence or absence of 1 µg/mL poly(I:C), 2 µM C16 and different concentrations of *eEF1A2* either in GDP-bound or GTP-bound conformations with 1 µM GDP or GppNHp, respectively. Mixtures were incubated at RT for 60 min and ADP detected with the ADP-Glo™ Kinase Assay System following the vendor’s instructions.

### Transactivation luciferase assay

NF-κB transactivation was assayed using the Bright-Glo™ Luciferase Assay System following the manufacturer’s instructions. MDA-MB-231 human breast cancer cells stably transfected with 3×NF-κB-tk-Luc plasmid (containing three NF-κB binding sites, a minimal promoter and a Renilla luciferase gene) were exposed to 50 ng/mL TNFα (positive control) or 450 nM plitidepsin for the indicated times. IκB kinase (IKK) inhibitors were used as controls, either alone or combined with TNFα or plitidepsin. Luminescence was measured in a Perkin-Elmer Victor3 reader (Waltham, MA, USA). A cell proliferation assay was simultaneously performed to control the cytotoxicity of the compounds.

### SPHK1 activity assay

SPHK1 activity was quantified with the Sphingosine Kinase Activity Assay Kit following the manufacturer’s instructions.

### S1P quantification

S1P levels were analysed with the S1P Competitive ELISA Kit following the manufacturer’s instructions.

### Ceramide quantification

Total lipid extracts from cell homogenates (2 × 10^6^ cells) were divided into three aliquots, two of them for the analysis of levels of sphingoid bases and the third for lipid phosphorus quantification. The first aliquot was hydrolysed for 1 h at 100 °C with 1 M KOH in methanol to deacylate ceramides and derivatives in total sphingoid bases. The second aliquot was used to determine the free sphingoid bases through mild alkaline hydrolysis at 37 °C (0.1 M KOH in chloroform–methanol 2:1 (v/v)). Long-chain bases from both aliquots were derivatised with *o*-phthalaldehyde, separated and quantified by high-performance liquid chromatography (HPLC) as described in ref.^15^ Results were expressed as picomoles of ceramide per nanomole of lipid phosphorus.

## Results

### Interaction of *eEF1A2* with PKR affects its catalytic activity

To identify new *eEF1A2*-interacting proteins, we performed immunoprecipitation experiments followed by a mass spectrometry-based proteomics approach. HeLa cells stably transfected with *eEF1A2*-tGFP were lysed and immunoprecipitated with an anti-tGFP Ab. As a control, wt HeLa cells were processed identically. After filtering for elongation factor isoforms, proteins involved in translation, common interacting actors such as chaperonins, proteins known to bind non-specifically to the immunoprecipitation matrix^16^ and proteins considered artefacts inherent to the experimental procedure (e.g. immunoglobulins), six proteins remained selected (Table 1). Our attention was drawn to PKR, a known regulator of cell survival in response to stress whose role in cancer has been unveiled in recent years.^17,18^

**Table 1:** Selected proteins identified after immunoprecipitation with anti-tGFP antibody in extracts of eEF1A2-tGFP HeLa cells

UNIPROT Accession number	Protein name (gene)	Score	%Coverage (95)^a^	Peptides (95%)
Q14166	Tubulin–tyrosine ligase-like protein 12 (*TTLL12*)	25.23	32.76	15
Q99714	3-Hydroxyacyl-CoA dehydrogenase type-2 (*HSD17B10*)	21.1	64.37	11
P19525	Interferon-induced, double-stranded RNA-activated protein kinase (*EIF2AK2*)	2.24	1.09	1
P07339	Cathepsin D (*CTSD*)	2.01	3.16	1
Q969S3	Zinc-finger protein 622 (*ZNF622*)	2	3.35	1
P51659	Peroxisomal multifunctional enzyme type 2 (*HSD17B4*)	1.85	1.9	1

^a^“% Coverage (95)” denotes the percentage of matching amino acids from identified peptides having confidence ≥95% divided by the total number of amino acids in the sequence, while “Peptides (95)” represents the number of distinct peptides having at least 95% confidence.

**Fig. 1:**
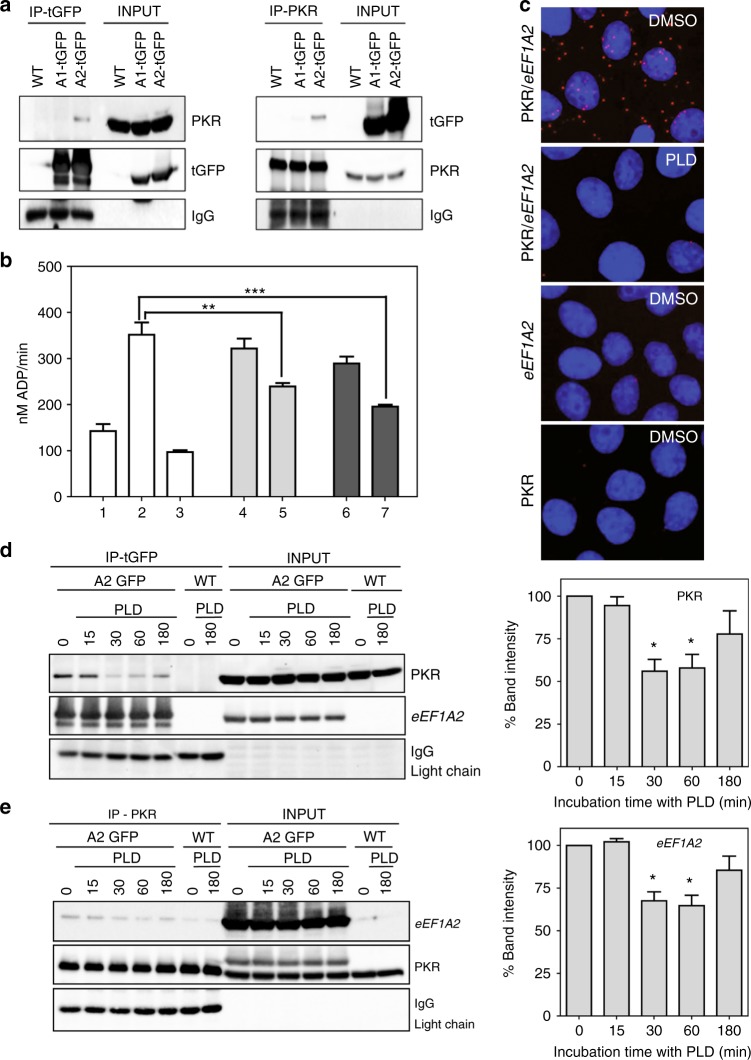
PKR inhibition by eEF1A2 and modulation of this protein–protein interaction by plitidepsin. **a** Whole extracts from wild-type (wt), eEF1A1-tGFP-transfected (A1-tGFP) or eEF1A2-tGFP-transfected (A2-tGFP) HeLa cells were processed for immunoprecipitation with antibodies against tGFP (first panel) or PKR (second panel). Co-immunoprecipitating proteins were detected by Western blotting using PKR or tGFP antibodies (first and second panels, respectively). IP-tGFP and IP-PKR represent the proteins immunoprecipitated with tGFP or with PKR antibodies. INPUT represents 10% of total immunoprecipitated protein before immunoprecipitation. The experiment was repeated three times with identical results and the pictures shown here are representative. **b** Effect of eEF1A2 on the kinase activity of PKR. The activity of human recombinant PKR was monitored using MBP as the substrate and measured by the release of ADP as described under Materials and Methods with 80 nM PKR alone (bar 1) or in the presence of 1 µg/mL poly(I:C) alone (bar 2) or together with either 2 µM C16 (bar 3), eEF1A2 in GDP-bound conformation at 80 or 160 nM (bars 4 and 5, respectively) or eEF1A2 in GTP-bound conformation at 80 or 160 nM (bars 6 and 7, respectively). Results correspond to one representative experiment out of three performed in 5-plicate with error bars denoting standard deviations. Comparison between different samples was analysed by Student’s *t* test. Differences were considered significant at *P < 0.05, **P < 0.01 and ***P < 0.001. **c** Proximity ligation assay (PLA) to detect by fluorescent microscopy the binding between eEF1A2 and PKR. HeLa wt cells were plated onto 16-well slides, treated with 0.1% DMSO (vehicle) or 450 nM PLD for 1 h and then stained following the instruction of the manufacturer. Cells were then photographed with a fluorescence microscope. **d**, **e** eEF1A2-GFP (A2-tGFP) HeLa or wild-type (wt) HeLa cell lines were treated with 450 nM PLD for the indicated times. Whole cell extracts were subjected to immunoprecipitation assays using anti-GFP antibodies (**d**) or PKR antibodies (**e**). Immunoprecipitates were subjected to SDS-PAGE and to immunoblotting assays using antibodies against PKR and tGFP. IP, total protein subjected to immunoprecipitation. INPUT, 10% total protein. The amount of eEF1A2/PKR complex was estimated through band densitometry. Averages of co-precipitated eEF1A2 and PKR were calculated from three different experiments and the values plotted in bar graphs beside each panel; error bars represent calculated standard errors. Comparison between different samples was analysed by Student’s *t* test. Differences were considered significant at *P < 0.05, **P < 0.01, ***P < 0.001

Since the interaction between *eEF1A2* and PKR had not been reported yet, we set out to confirm this result. Extracts from HeLa cells expressing chimeric *eEF1A2*-tGFP or eEF1A1-tGFP constructs were immunoprecipitated with either anti-GFP or anti-PKR antibodies. PKR was detected in the anti-GFP-immunoprecipitated material from *eEF1A2*-tGFP-expressing HeLa cells, but not in the anti-GFP-immunoprecipitated material from eEF1A1-tGFP-expressing HeLa cells (Fig. 1a). Similarly, *eEF1A2*-GFP, but not eEF1A1-tGFP, was identified in the PKR-immunoprecipitated sample (Fig. 1a), demonstrating the specificity of the binding between PKR and *eEF1A2*. Immunoprecipitation of extracts from HeLa cells ectopically overexpressing human *eEF1A2*-tGFP, as well as myc-tagged human PRDX1, SPHK1 or SPHK2, confirmed the previously described interaction between these three proteins and *eEF1A2* (Supplementary Figures 1, 2A).

To understand whether the kinase function of PKR was affected by its interaction with *eEF1A2*, we measured the activity of the recombinant human enzyme produced by an in vitro translation system. Figure 1b shows that the kinase was functionally active and its activity was boosted by the viral dsRNA mimetic poly(I:C) and abated by the specific PKR inhibitor C16.^19^ Interestingly, the presence of rabbit *eEF1A2* caused an inhibitory effect on the kinase activity of PKR, more pronounced when the PKR–*eEF1A2* molar ratio was 1:2 (*p* < 0.005). This inhibition was observed for both nucleotide-dependent conformations of *eEF1A2*, although the GTP-bound form was slightly more efficient. Taken together, these results demonstrate that *eEF1A2* directly interacts with PKR and impairs its catalytic activity.

### *eEF1A2*–PKR complex formation inhibits the pro-apoptotic activity of PKR

The observation of *eEF1A2*–PKR complexes prompted us to investigate their biological role in tumour cells. We first examined whether targeting of *eEF1A2* by plitidepsin was able to modulate *eEF1A2*–PKR complex formation in HeLa cells. Taking advantage of the PLA, we observed by fluorescent microscopy the formation of *eEF1A2*–PKR complexes within HeLa wt cells (Fig. 1c, upper panel). These complexes were lost when cells were incubated for 1 h with 450 nM plitidepsin (Fig. 1c, second panel). Controls lacking each of the primary antibodies used for the assay are shown in the third and fourth panels.

Wt and *eEF1A2*-tGFP-transfected HeLa cells were treated for different times with 450 nM plitidepsin and cell lysates subjected to immunoprecipitation. Plitidepsin treatment resulted in a rapid and sustained reduction of *eEF1A2*–PKR complexes, as deduced by the disappearance of the anti-PKR Ab immunoreactive band in the anti-tGFP-immunoprecipitated material (Fig. 1d) or the anti-GFP Ab immunoreactive band in the anti-PKR-immunoprecipitated material (Fig. 1e). Moreover, plitidepsin also disrupted, in a time-dependent manner, *eEF1A2*–PRDX1 complexes (Supplementary Fig. 1). Of note, the interaction between *eEF1A2* and both SPHK1 and SPHK2 was not affected by the drug (Supplementary Figs. 2, 3).

**Fig. 2:**
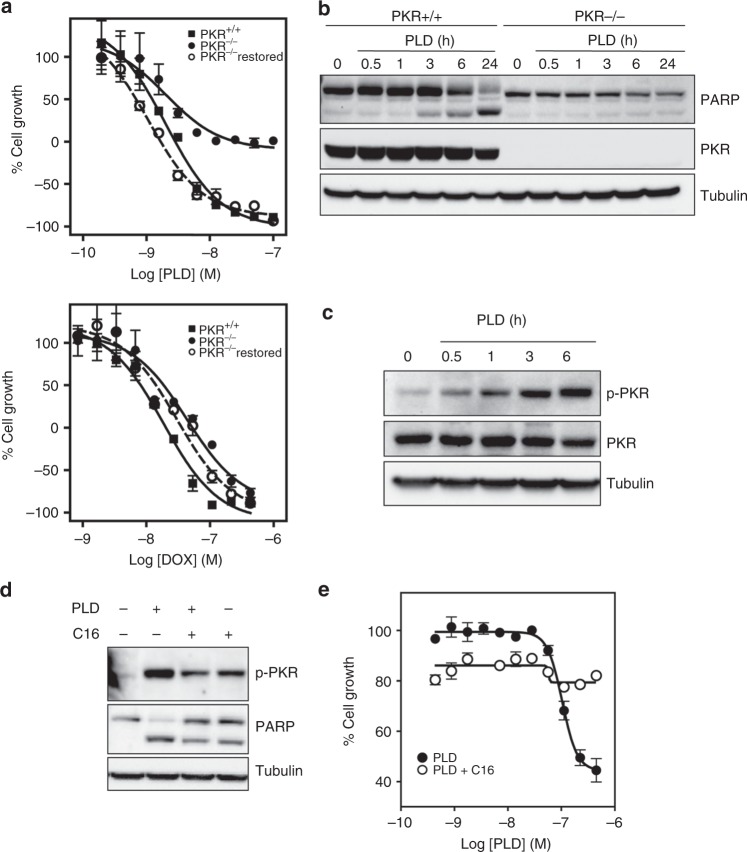
PKR involvement in the antiproliferative effect of plitidepsin. **a** Wild-type (PKR^+/+^) (closed square), PKR^−/−^ (closed circle) and PKR^−/−^ restored for PKR (open circle) MEFs were exposed to plitidepsin (PLD) or doxorubicin (DOX) at several concentrations for 72 h. Cell growth was determined by the MTT method and expressed as the percentage of control cell growth. **b** PKR^+/+^ and PKR^−/−^ MEFs were treated with 450 nM PLD for the indicated time points. PARP cleavage was analysed by Western blot. Membranes were re-probed with antibodies against total PKR and tubulin for normalisation. **c** HeLa cells were treated with 450 nM PLD for the indicated time points and the phosphorylation status of PKR was analysed by Western blot. Membranes were re-probed with antibodies against total PKR and tubulin. **d** HeLa cells were pre-incubated with vehicle (−) or 100 µM C16 (+) for 60 min before being treated with 450 nM PLD (+) for 6 h. Phosphorylated form of PKR was analysed by Western blot. Membranes were re-probed with antibodies against PARP and tubulin for normalisation. **e** Growth inhibition curves for HeLa cells after 6 h of exposure to several PLD (closed circle) and C16 + PLD (open circle) concentrations. Due to the short duration of the co-treatment experiment, cell growth was determined by the SRB method

To gain further insight into the biological effects of plitidepsin-induced *eEF1A2*–PKR complex disruption, we evaluated the sensitivity to the drug of PKR^−/−^, PKR^−/−^ with re-expressed PKR and wt MEFs. As observed in Fig. 2a (upper panel), sensitivity to plitidepsin was significantly reduced in PKR^−/−^ MEFs and recovered to the levels shown by wt MEFs when PKR was ectopically re-expressed in the null fibroblasts, hence suggesting a critical role for PKR in the antiproliferative effect of this drug after binding to *eEF1A2*. To notice, the effect of a control compound (doxorubicin, DOX) was not affected by the presence of PKR (Fig. 2a, lower panel). Indeed, PKR appears essential for leading MEFs into apoptosis upon plitidepsin treatment as evidenced by the absence of PARP processing in drug-treated PKR^−/−^ cells (Fig. 2b). To confirm these effects in tumour cells, we explored whether plitidepsin induced the activation of PKR, as measured by its self-phosphorylation on Thr-451, in HeLa cells. Figure 2c demonstrates that treatment of HeLa cells with 450 nM plitidepsin elicited a sustained increase in the levels of autophosphorylated PKR, while the total kinase levels remained unaffected. However, such phosphorylation was significantly reduced when HeLa cells had been pretreated with the PKR inhibitor C16 before plitidepsin exposure (Fig. 2d). Moreover, reduction in PKR self-phosphorylation caused by co-treatment with C16 was concomitant with a decrease in the ability to induce PARP processing, hence supporting the essential role of PKR in plitidepsin-promoted apoptosis. To corroborate this conclusion, the antiproliferative effect of plitidepsin on HeLa cells was evaluated in the presence and absence of C16. Figure 2e demonstrates that, indeed, C16 cotreatment protected HeLa cells from the lethal effect of plitidepsin, clearly evidencing the critical role of PKR in the mechanism of action of the drug. Altogether, these results show that the presence of *eEF1A2*–PKR complexes is necessary to inhibit the pro-apoptotic functions of PKR in tumour cells.

### Inhibition of *eEF1A2* with plitidepsin induces apoptosis dependent on PKR activation

**Fig. 3:**
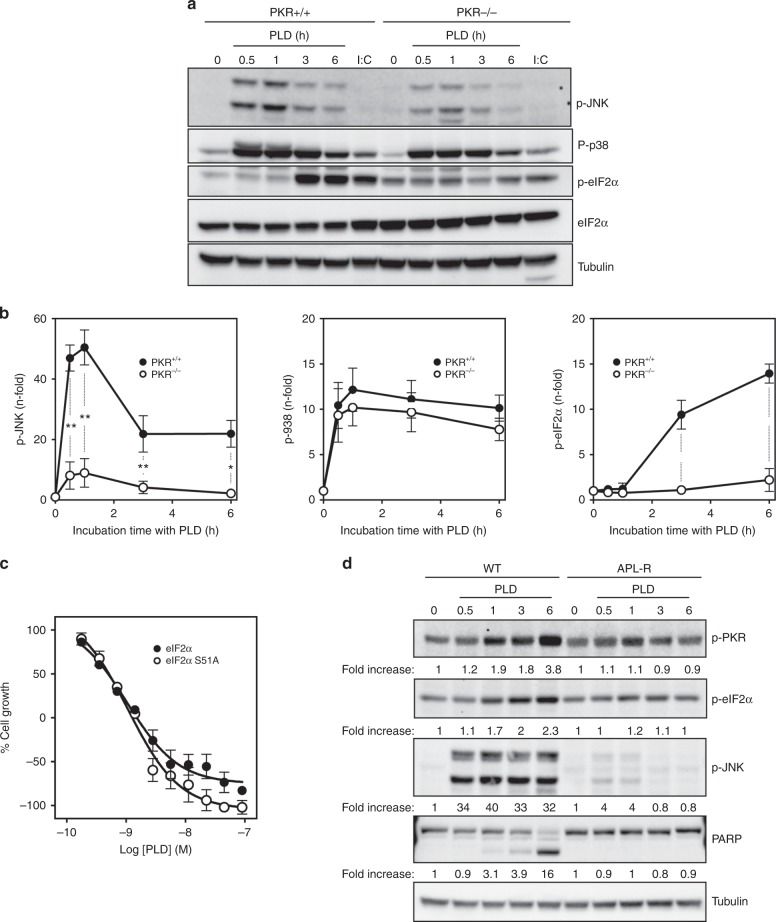
Sustained activation of JNK by plitidepsin is partially dependent on PKR activation. **a** Wild-type (PKR^+/+^) and PKR^−/−^ MEFs were treated with 450 nM PLD for the indicated time points (in h) and the phosphorylation status of JNK, p38 and eIF2α were analysed by Western blot. Poly(I:C) (polyinosinic:polycytidylic acid) was used as a control inducer of eIF2α phosphorylation. Membranes were re-probed with antibodies against total eIF2α and tubulin. **b** The intensities of the bands were quantified, and the values, expressed as *n*-fold increases with respect to the untreated control cells, were plotted in graphs showing the results for wild-type (closed circle) and PKR^−/−^ (open circle) MEFs. Differences were considered significant at *P < 0.05, **P < 0.01 and ***P < 0.001. **c** Cell growth inhibition curves for MEFs expressing wild-type eIF2α (closed circle) or eIF2α S51A (open circle). **d** WT and APL-R HeLa cells were treated with 450 nM plitidepsin for the indicated times. PARP cleavage and phosphorylated forms of PKR, eIF2α and JNK were analysed by Western blotting. Membranes were re-probed with antibodies against total tubulin. Numbers refer to fold-increase activation with respect to untreated cells (lanes marked as “0”). The experiment was repeated three times with similar results

As mentioned above, targeting of *eEF1A2* with plitidepsin induces the activation of proteins involved in pro-apoptotic signalling such as JNK and p38/mitogen-activated protein kinase (MAPK). Figure 3a, b show the rapid activation of these proteins in wt MEFs treated with 450 nM plitidepsin, the highest activation reached between 0.5 and 1 h post-treatment. However, PKR removal in these cells led to a significantly weaker activation of JNK. Likewise, plitidepsin induced the rapid and sustained phosphorylation of eIF2α in wt MEFs but not in PKR^−/−^ cells. The lack of eIF2α phosphorylation in these latter cells, even in the presence of the PKR activator poly-I:C,^20^ confirmed the null expression of PKR in this cell line (Fig. 3a). Interestingly, although poly-I:C effectively promoted eIF2α phosphorylation in wt MEFs, it did not promote per se JNK or p38 activation. These findings clearly point to a unique involvement of PKR in the plitidepsin-induced activation of these signalling kinases. Nonetheless, despite the essential role of PKR in the apoptotic effect of plitidepsin, PKR-dependent phosphorylation of eIF2α seemed not to be critical for plitidepsin lethality. Indeed, MEFs expressing a non-phosphorylatable eIF2α (eIF2αS51A) were as sensitive to plitidepsin as wt MEFs (Fig. 3c). In addition, treatment of these mutant MEFs (completely lacking eIF2α phosphorylation, Supplementary Fig. 4) with plitidepsin induced the same pro-apoptotic signalling events observed in wt cells, namely rapid JNK activation and later PARP processing (Supplementary Fig. 4).

We have finally checked the activation pattern of PKR in plitidepsin-resistant tumour cells with reduced *eEF1A2* protein levels.^9^ Treatment with 450 nM plitidepsin induced a clear, time-dependent increase in phosphorylated PKR in HeLa cells (Fig. 3d). However, in *eEF1A2*-deficient cells (HeLa-APL-R), phospho-PKR levels did not change significantly after plitidepsin treatment. These differences in PKR self-phosphorylation, mirrored in the levels of phosphorylated eIF2α, were parallel to differences in the signature of the drug in both HeLa strains, that is, JNK activation and PARP processing, which were visible in wt HeLa cells but not in the resistant ones (Fig. 3d). This result confirmed the essential role of PKR in the apoptosis induced after plitidepsin binding to *eEF1A2*.

### *eEF1A2*–PKR complexes regulate the NF-κB signalling pathway

**Fig. 4:**
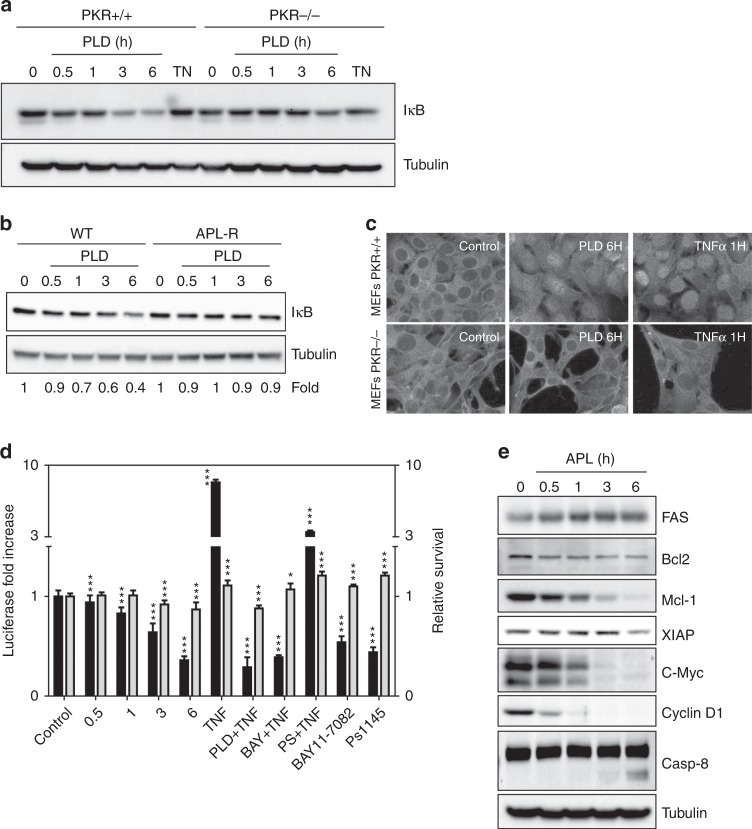
PKR-mediated, plitidepsin-induced triggering of the pro-apoptotic activity of NF-κB while inhibiting its pro-survival network. **a** Wild-type (PKR^+/+^) and PKR^−/−^ MEFs were treated with 450 nM PLD for the indicated times or, as a positive activation control, 50 ng/mL tunicamycin (“TN”). IκB protein levels, as NF-κB pathway status indicator, were analysed by Western blot. Tubulin was analysed as loading control. **b** Wild-type and plitidepsin-resistant (“APL-R”) HeLa cells were treated with 450 nM plitidepsin (PLD) for the indicated times and IκB protein levels analysed as above. **c** Wild-type (PKR^+/+^) and PKR^−/−^ MEFs were treated with 450 nM plitidepsin (PLD) for 6 h or 50 ng/mL TNFα for 1 h, fixed with 4% paraformaldehyde and hybridised with an anti-NF-κB primary antibody and an Alexa 488-labelled secondary antibody. Cells were then photographed with a fluorescence microscope. **d** MDA-MB-231 cells stably transfected with 3 × NF-κB-tk-Luc plasmid were treated with 450 nM PLD for the indicated times, 50 ng/mL TNFα (as positive activation control), 100 µM BAY11-7082 or 20 µM PS1145 (both IKK inhibitors), or combinations thereof. Luciferase expression levels (black bars) were then analysed by luminometry using the GloMax Kit (Promega). Survival (white bars) was analysed with the MTT assay. Differences were considered significant at *P < 0.05 and ***P < 0.001. **e** Expression of relevant NF-κB target genes was analysed by Western blot in HeLa cells treated with 450 nM PLD for the indicated times

We then checked the effect of targeting *eEF1A2* by plitidepsin on the NF-κB signalling pathway, which is involved in the downstream pro-apoptotic effect of PKR.^21^ As a marker of NF-κB activation, we first investigated whether IκB was phosphorylated by IKK and, thus, degraded by the proteasome following plitidepsin treatment. Indeed, wt MEFs treated with 450 nM plitidepsin showed a clear IκB degradation after 3 h, absent in PKR^−/−^ MEFs (Fig. 4a). To ensure that NF-κB activation also occurred in cancer cells in an *eEF1A2*-dependent fashion, wt and APL-R HeLa cells were treated with 450 nM plitidepsin for the indicated times and the levels of IκB checked by Western blot. While in wt HeLa cells plitidepsin induced a clear degradation of IκB, in APL-R HeLa cells, lacking *eEF1A2*, no alteration of IκB levels was observed (Fig. 4b). Then, we analysed by immunofluorescence the cellular localisation of NF-κB in wt or PKR^−/−^ MEFs treated with plitidepsin 450 nM for 6 h or, as a positive activation control, with 50 ng/mL of TNFα for 1 h (Fig. 4c). NF-κB shuttled to the nucleus in wt MEFs after treatment with either TNFα or plitidepsin, demonstrating the activation of the pathway. In contrast, in PKR^−/−^ MEFs we could not see any shuttling of NF-κB after TNFα or plitidepsin treatments, indicating that the activation observed in wt MEFs was dependent on PKR.

Since NF-κB localised to the nuclei in plitidepsin-treated wt MEFs, we checked whether the transcriptional activity of the protein was also activated. To that end, we took advantage of MDA-MB-231 cells stably transfected with an NF-κB luciferase reporter plasmid. We treated the cells with either 450 nM plitidepsin for times ranging from 0.5 to 6 h, 50 ng/mL TNFα (an activator of NF-κB), 100 µM BAY11-7082, 20 µM PS1145 (both IKK inhibitors) or combinations of them and quantified the luciferase activity under each condition. Even in the presence of 50 ng/mL TNFα, plitidepsin clearly inhibited the production of luciferase indicating that transactivation from NF-κB was inhibited in the presence of the drug (Fig. 4d). Taken together, these findings demonstrate that NF-κB activation by plitidepsin was dependent on the presence of *eEF1A2* and its interaction with PKR.

We then checked whether the expression of some NF-κB-responsive genes was affected by plitidepsin. HeLa cells were treated with 450 nM plitidepsin for times ranging from 0.5 to 6 h and the cellular levels of FAS, Bcl2, Mcl1, XIAP, c-myc and cyclin D1 were analysed by Western blot. Plitidepsin clearly inhibited the expression of Bcl2, Mcl1, XIAP, c-myc and cyclin D1, while FAS expression was activated (Fig. 4e).

### *eEF1A2*–PKR complexes regulate the extrinsic apoptotic pathway

**Fig. 5:**
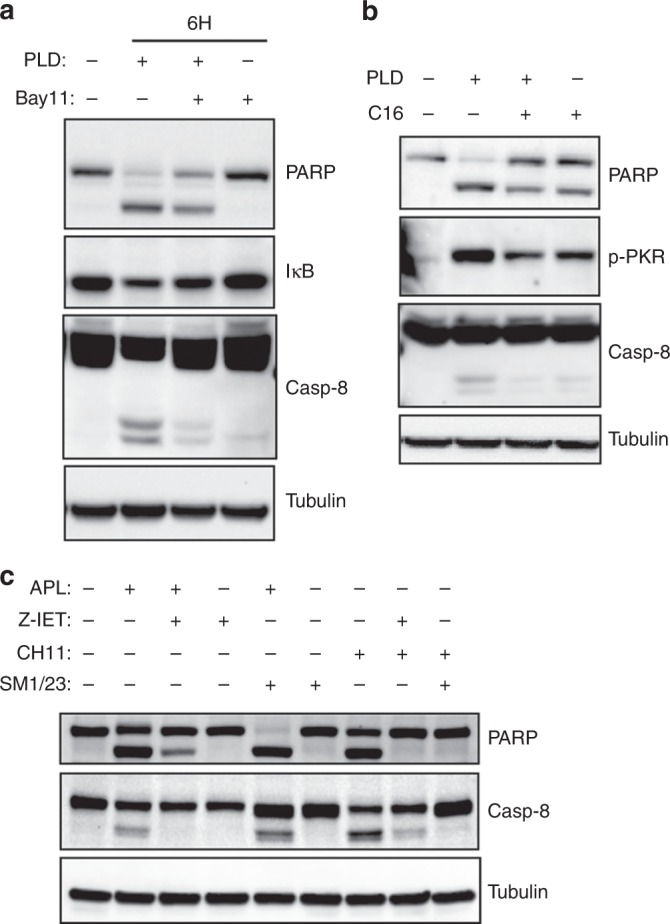
Plitidepsin triggers the extrinsic apoptotic pathway in a PKR-dependent, NF-κB-dependent and Fas-dependent way. HeLa cells were treated with 450 nM PLD and/or 100 µM Bay11-7082 for 6 h. PARP1 cleavage, as a marker of apoptosis, IκB, as a marker of NF-κB activation, and caspase-8 cleavage, as a marker of extrinsic apoptosis, were analysed by Western blot. **b** HeLa cells were treated with 450 nM PLD, 100 µM C16 (PKR inhibitor) or a mixture of both for 6 h. Levels of caspase-8 cleavage, PARP1 processing and phospho-PKR were analysed by Western blot. **c** HeLa cells were treated for 6 h with 450 nM PLD and/or the indicated amounts of Z-IETD (µM), 0.5 µg/mL CH11 Fas-activating antibody or 1 µg/mL SM1/23 Fas-blocking antibody for 6 h and cleavage of caspase-8, indicating the activation of the extrinsic apoptotic pathway, and PARP1 processing, indicating apoptotic death, were analysed by Western blot

After these results, we were interested in learning whether NF-κB activation was necessary for plitidepsin-induced apoptosis. HeLa cells were treated with 450 nM plitidepsin, 100 µM BAY11-7082 or a combination of both for 6 h and PARP1 cleavage was analysed as apoptosis marker. Co-treatment with plitidepsin and the IKK inhibitor BAY11-7082 clearly reduced the pro-apoptotic effect of the drug, showing that NF-κB activation was indeed necessary for plitidepsin-induced apoptosis (Fig. 5a). Fas signalling has been shown to be activated by NF-κB at the transcriptional level^22,23^ and PKR-induced apoptosis depends on the activation of Fas by NF-κB.^21,24^ Noteworthy, plitidepsin has been shown to induce the extrinsic apoptotic pathway in Jurkat leukaemia cells.^25,26^ Thus, we analysed whether plitidepsin was activating the extrinsic apoptotic pathway also in HeLa cells. Caspase-8 was, indeed, activated by auto-cleavage in plitidepsin-treated HeLa cells, and co-treatment with the IKK inhibitor BAY11-7082 partially inhibited this activation (Fig. 5a).

To check whether the NF-κB-dependent apoptotic activation induced by plitidepsin was dependent on PKR, we treated HeLa cells with 450 nM plitidepsin, 100 µM C16 (PKR inhibitor) or a mixture of both for 6 h and checked by Western blot the levels of PKR phosphorylation, caspase-8 activation and PARP1 cleavage. Inhibition of PKR with C16 blocked both caspase-8 activation and PARP1 cleavage clearly indicating that plitidepsin-induced apoptosis was dependent on PKR (Fig. 5b). Finally, we explored whether the activation of the extrinsic apoptotic pathway by plitidepsin was due to Fas clustering (as previously demonstrated in Jurkat cells^26^) or through other external stimuli (e.g. FasL). We treated HeLa cells for 6 h with 450 nM plitidepsin, 50 µM caspase-8 inhibitor Z-IETD, 0.5 µg/mL Fas-activating Ab CH11 or 1 µg/mL Fas-inhibiting Ab SM1/23 (or combinations thereof, pre-incubating 1 h with the inhibitors Z-IETD or SM1/23 when present) and checked caspase-8 activation. Plitidepsin-induced caspase-8 activation was specifically inhibited by co-treatment with Z-IETD. Furthermore, co-treatment with SM1/23 did not reduce the pro-apoptotic effect of plitidepsin, indicating that the effect of this drug was likely due, at least in part, to the induction of Fas clustering rather than pathway activation by FasL (Fig. 5c).

## Discussion

For a long time, human *eEF1A2* has been known to behave as a pro-oncogenic protein, its aberrant expression increasing cancer cell fitness through the inhibition of apoptosis,^12^ control of misfolded proteins degradation,^11^ heat-shock response,^27^ reorganisation of actin cytoskeleton^10^ and regulation of oxidative stress.^8^ Several interaction partners have been described for *eEF1A2* that are important to convey its pro-oncogenic properties, including PRDX1,^8^ SPHK1^6,7^ and others. We have recently demonstrated that *eEF1A2* is the main molecular target of plitidepsin, a marine drug currently in development for the treatment of multiple myeloma patients.^9^ Here we show that PKR is a novel *eEF1A2*-interacting partner, whose pro-apoptotic activity is regulated by the elongation factor likely through the inhibition of its kinase activity. Targeting of *eEF1A2* with plitidepsin releases PKR from the complex, facilitating its activation and, in doing so, it triggers the MAPK-dependent and NF-κB-dependent activation of the extrinsic apoptotic pathway. Our data stress the important role of the “moonlighting” effects of *eEF1A2* in the maintenance of cancer phenotype.

PKR is a dsRNA-dependent serine/threonine protein kinase better known for its role in the innate immune response against viral infections.^28^ PKR can also be activated, in a dsRNA-independent manner, through the PKR-associated activator (PACT) in response to oxidative stress (H_2_O_2_)^29^ or the second messenger ceramide.^30^ Given that PKR functions as a tumour suppressor controlling cell growth and proliferation,^17,31,32^ it can be considered a good target for cancer therapy, the rationale being selectively activating the kinase to trigger tumour cell apoptosis.^17^ Herein, we show that *eEF1A2* binds to PKR, most likely keeping it inactive since *eEF1A2* reduces the phosphorylation of MBP catalysed by PKR. Remarkably, upon plitidepsin binding to *eEF1A2*, PKR is released from this complex and becomes activated, probably through several cellular stimuli such as oxidative stress or increased ceramide levels. Indeed, through its binding to *eEF1A2*, plitidepsin could actively generate both oxidative stress, by decreasing PRDX1 activity, and increased ceramide levels, inhibiting SPHK1 and displacing the sphingolipid equilibrium towards ceramide synthesis. Once activated, PKR is a potent apoptotic inducer.^17^

Induction of apoptosis by PKR has been related to the phosphorylation of eIF2α,^33^ the activation of MAPKs (JNK and p38)^34^ or the overexpression of Fas (CD95/Apo-1), a TNFR family member,^24^ through transactivation by NF-κB. We now show that plitidepsin binding to *eEF1A2* does, indeed, induce hyper-phosphorylation of eIF2α in a PKR-dependent fashion, although this phosphorylation seems to be irrelevant for the antiproliferative activity of the compound given that non-phosphorylatable eIF2αS51A homozygous MEFs are as sensitive to the drug as wt MEFs. MAPKs p38 and JNK have been shown to be activated by plitidepsin, JNK being essential for the apoptotic effect of this drug.^35^ Here we show that JNK activation by plitidepsin is, at least in part, dependent on the disruption of *eEF1A2*–PKR complexes, since PKR-deficient fibroblasts have diminished JNK activation and apoptosis in response to the compound. Furthermore, in HeLa-APL-R cells lacking *eEF1A2*, plitidepsin was unable to induce PKR self-phosphorylation and, therefore, JNK activation was also greatly diminished. Thus, signalling through JNK is important for the apoptogenic effect of plitidepsin-activated PKR.

A third way to execute PKR-induced apoptosis is the NF-κB signalling pathway. This may appear paradoxical since, through the transactivation of a plethora of anti-apoptotic genes, NF-κB is widely accepted as one of the main pro-survival pathways in the cell.^36^ Nevertheless, there is also evidence on the pro-apoptotic effect of the NF-κB pathway, through the transactivation of pro-apoptotic genes such as *Fas*, *FasL*, *TRAIL*, *p53* or *Bcl-xS* (reviewed in ref.^37^) We now report that plitidepsin, in a PKR-dependent and *eEF1A2*-dependent way, activates the NF-κB pathway, inducing the degradation of IκB and the nuclear translocation of NF-κB. Furthermore, plitidepsin-induced apoptosis was partially dependent on NF-κB activation, since pretreatment of HeLa cells with the IKK inhibitor BAY11-7082 clearly diminished cell death. We show that plitidepsin blocks TNFα-induced luciferase transactivation in MDA-MB-231 cells transfected with an NF-κB-responsive luciferase reporter plasmid. Moreover, during plitidepsin treatment in HeLa cells, most NF-κB-transactivated genes, especially those involved in growth and survival such as c-myc,^38^ cyclin D1,^39^ Bcl2,^40^ Mcl1^41^ or XIAP^42^ were down-regulated, with the exception of pro-apoptotic Fas (CD95)^22,23,43^ whose expression was boosted. Fas has been shown to be involved in the pro-apoptotic effect of plitidepsin in human leukaemic cells.^25,26^ Here we demonstrate that caspase-8 is activated by plitidepsin in HeLa cells, concomitant to an induction of apoptosis that can be suppressed with the IKK inhibitor BAY11-7082 or with the PKR inhibitor C16. Thus, though NF-κB is acknowledged for the transactivation of pro-survival genes, activation of the pathway by plitidepsin is essential for the induction of apoptosis in cancer cells. Fas clustering, rather than binding to its ligand FasL, has been previously shown to activate the extrinsic apoptotic pathway in Jurkat T cell leukaemia cells treated with plitidepsin.^26^ We now extend this discovery to other cell types, demonstrating that HeLa cells treated with plitidepsin activate the extrinsic apoptotic pathway through Fas clustering, by NF-κB transactivation of the receptor, rather than through lipid raft modification.

From a structural viewpoint, the best-characterised binding partners for PKR are eIF2α^44^ and the vaccinia virus protein K3L, which mimics the 3D structure of eIF2α and its mode of interaction with PKR, thereby competitively blocking eIF2α phosphorylation on Ser51.^45^ The GTP-binding pocket of eIF2 is contained within the γ-subunit and the guanine nucleotide exchange factor for eIF2 is eIF2B,^46^ which exchanges GDP for GTP on the γ-subunit of eIF2 and is inhibited by stress-induced phosphorylation of eIF2α.^47^ This α-subunit of eIF2 consists of two domains,^48^ the C-terminal one being structurally very similar to the C-terminal domain of eEF1Bα, that is, the α-subunit of the guanine nucleotide exchange factor for *eEF1A2*.^49^ Thus, structural and functional similarities exist between eIF2α–eIF2γ and eEF1Bα–eEF1A pairs.^48^ Interestingly, the crystal structure of *eEF1A2* from rabbit muscle in complex with GDP revealed a tightly bound homodimer in the asymmetric unit,^50^ whereas the complex between eIF2α and the catalytic domain of PKR disclosed a binding mode that nicely accounts for the accessibility of the (missing) loop containing Ser51 into the catalytic cleft of PKR.^44^ A non-classical pathway leading to the full catalytic activation of PKR is triggered by direct interaction with PACT/RAX, a cellular protein that can heterodimerise directly with PKR.^51^ It seems feasible that dissociation of the *eEF1A2* homodimer from PKR can release the intramolecular auto-inhibition effected by the dsRNA-binding domains and lead to activation of the kinase domain and the observed eIF2α phosphorylation.

**Fig. 6:**
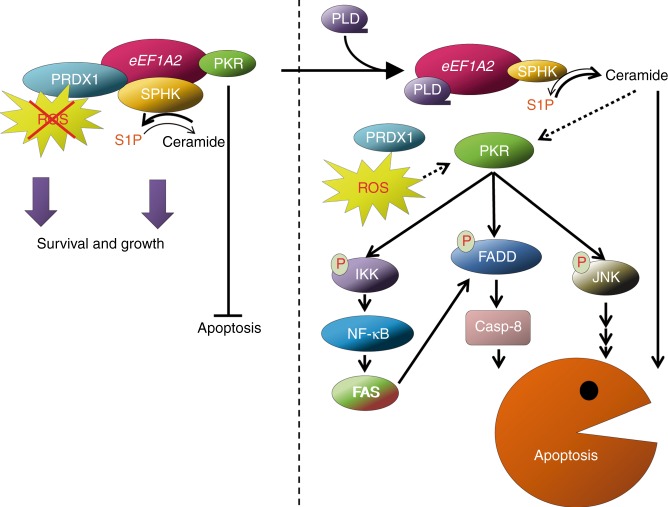
Proposed pro-oncogenic mechanism of eEF1A2 and its inhibition by plitidepsin. When expressed in cancer cells, eEF1A2 forms complexes with several partners that elicit the pro-oncogenic activities of the elongation factor. Binding to PRDX1 and SPHK, eEF1A2 potentiates their activity, thus reducing the oxidative stress and increasing the S1P production within the cancer cell, providing it with improved growth and survival properties. Furthermore, sequestering PKR, eEF1A2 inhibits its pro-apoptotic activity. When cancer cells are treated with plitidepsin, the interaction between eEF1A2 and PRDX1 is broken, therefore reducing the antioxidant capacity of PRDX1 and increasing oxidative stress. SPHK is not released from its complex with eEF1A2 by plitidepsin, but the activity of the lipid kinase is greatly reduced, diminishing the cellular level of S1P and unbalancing the equilibrium towards the formation of pro-apoptotic ceramides. Plitidepsin also released PKR from its complex with eEF1A2, rendering the kinase active (either directly or through the stress stimuli generated by the compound). Active PKR, on its turn, phosphorylates downstream targets as JNK or FADD with clear pro-apoptotic activity. Furthermore, although PKR also phosphorylates IKK and releases NF-κB from its inhibitor IκB, it seems that the transcription factor is unable to activate pro-survival genes but transactivates FAS, the death receptor that has been previously shown to trigger the apoptotic effect of plitidepsin. The final outcome of the inhibition of the pro-oncogenic properties of eEF1A2 by plitidepsin is the death of cancer cells

In summary, our findings shed light on several moonlighting functions of *eEF1A2* (proposed mechanism in Fig. 6), which support the pro-oncogenic properties of this alternative elongation factor, and uncover PKR as a new protein binding partner whose regulation may be essential for the inhibition of apoptosis. It is tempting to think of *eEF1A2* as an essential cell death regulator, necessary for the survival of tissues with low rates of cell division after embryonic development, mostly in brain, heart and muscle. This would be the reason why embryonic eEF1A1 expression in those tissues is switched off in the adult and replaced by *eEF1A2*: the A2 homologue would inhibit apoptosis in tissues with low cell renewal, helping them to maintain their special homeostasis. In cancer cells, aberrant *eEF1A2* expression would provide resistance to cell death, an essential advantage that would help tumour cells to overcome their intrinsic weaknesses, allowing them to grow. Plitidepsin, through its binding to *eEF1A2*, would release the apoptotic brake imposed by this elongation factor, exposing the frailty of the tumour cells, especially when compared with healthy tissues expressing *eEF1A2*, and leading them to a PKR-dependent apoptotic cell death. The fitness boost that these *eEF1A2* non-canonical functions provide to cancer cells would then be important for their growth and survival. All in all, *eEF1A2* emerges as an Achilles heel in tumour cells that can be targeted for anticancer therapy.

## Electronic supplementary material

Supplementary Figure 1

Supplementary Figure 2

Supplementary Figure 3

Supplementary Figure 4

Supplementary Figure 5

## Availability of data and material

Material, data and associated protocols are available to readers upon request.
