# Supplementary figures and images for: Binding of eEF1A2 to the RNA-dependent protein kinase PKR modulates its activity and promotes tumour cell survival
Source: Br J Cancer. 2018 Nov 13;119(11):1410–20. doi: 10.1038/s41416-018-0336-y (PMC6265344; doi:10.1038/s41416-018-0336-y)

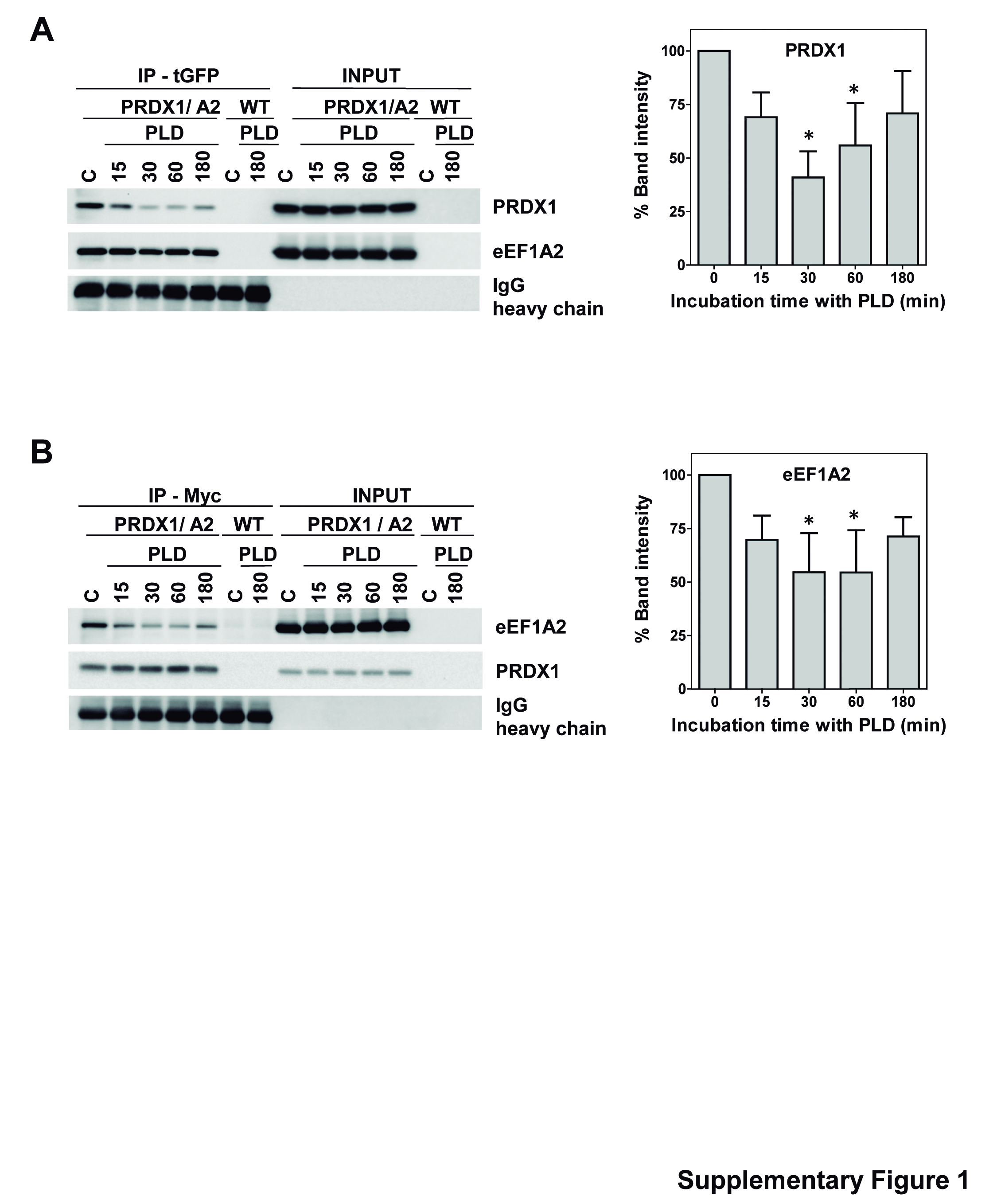

Supplement: Supplementary file 1 — Supplementary Figure 1 [file 41416_2018_336_MOESM1_ESM.tif]

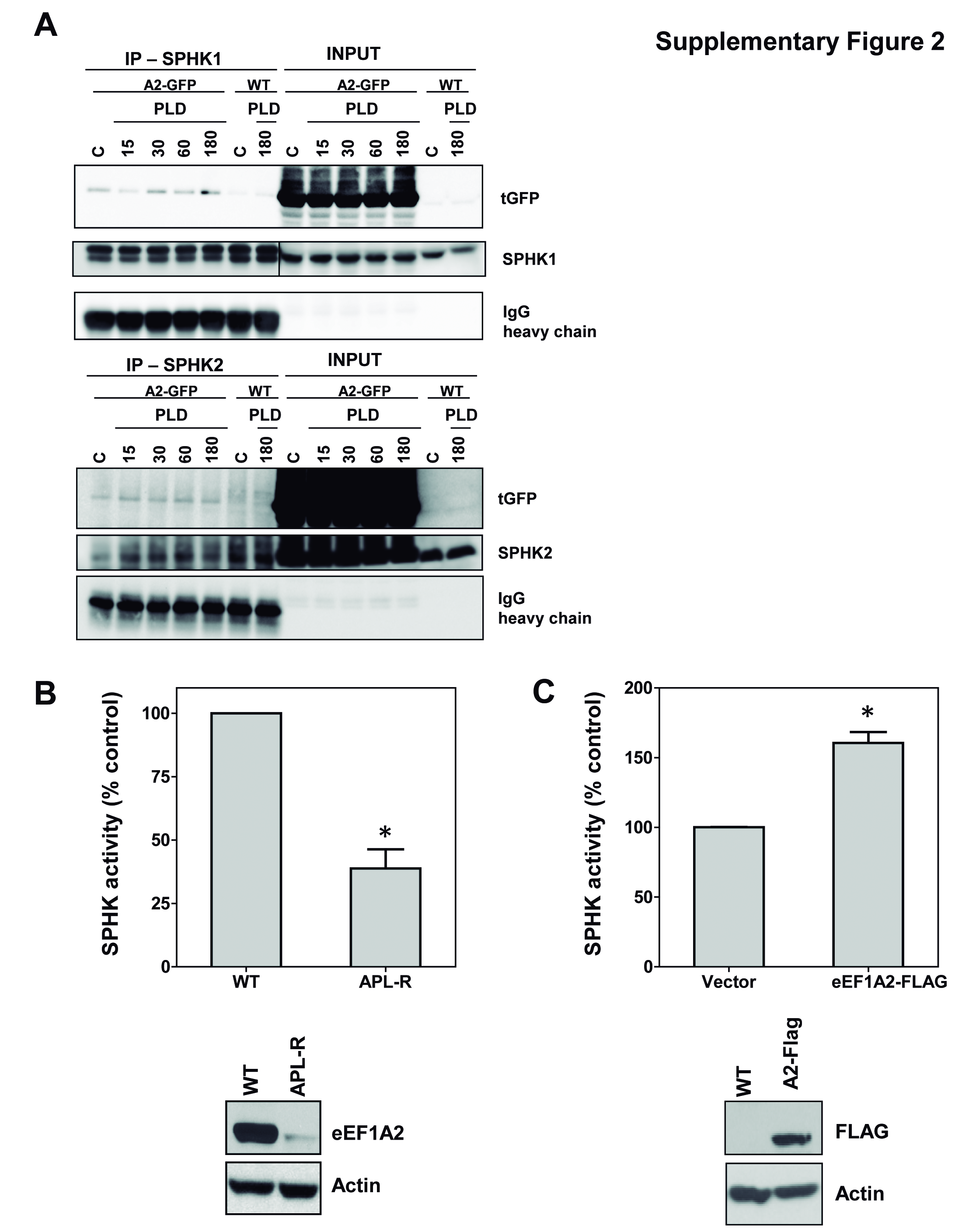

Supplement: Supplementary file 2 — Supplementary Figure 2 [file 41416_2018_336_MOESM2_ESM.tif]

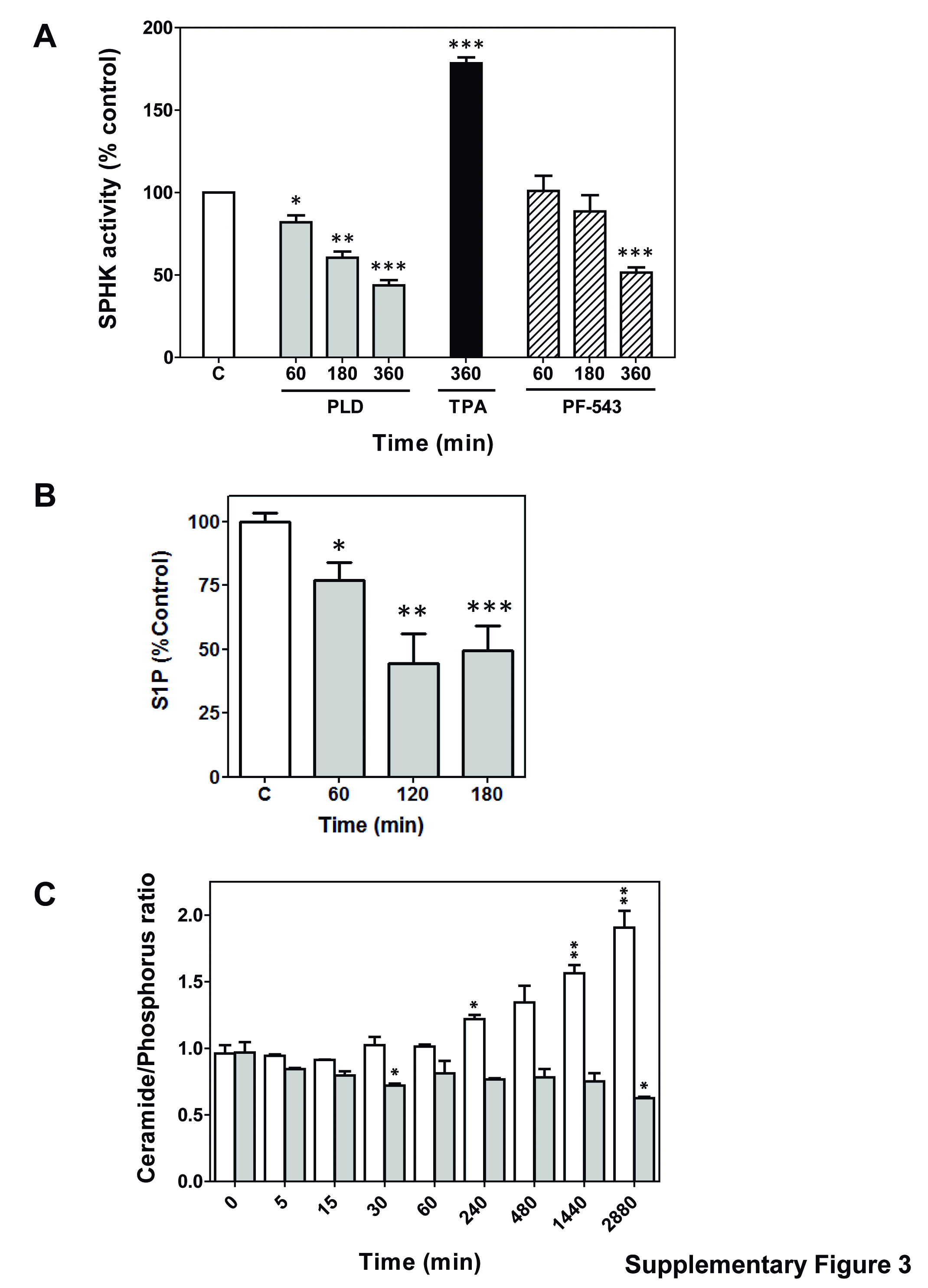

Supplement: Supplementary file 3 — Supplementary Figure 3 [file 41416_2018_336_MOESM3_ESM.tif]

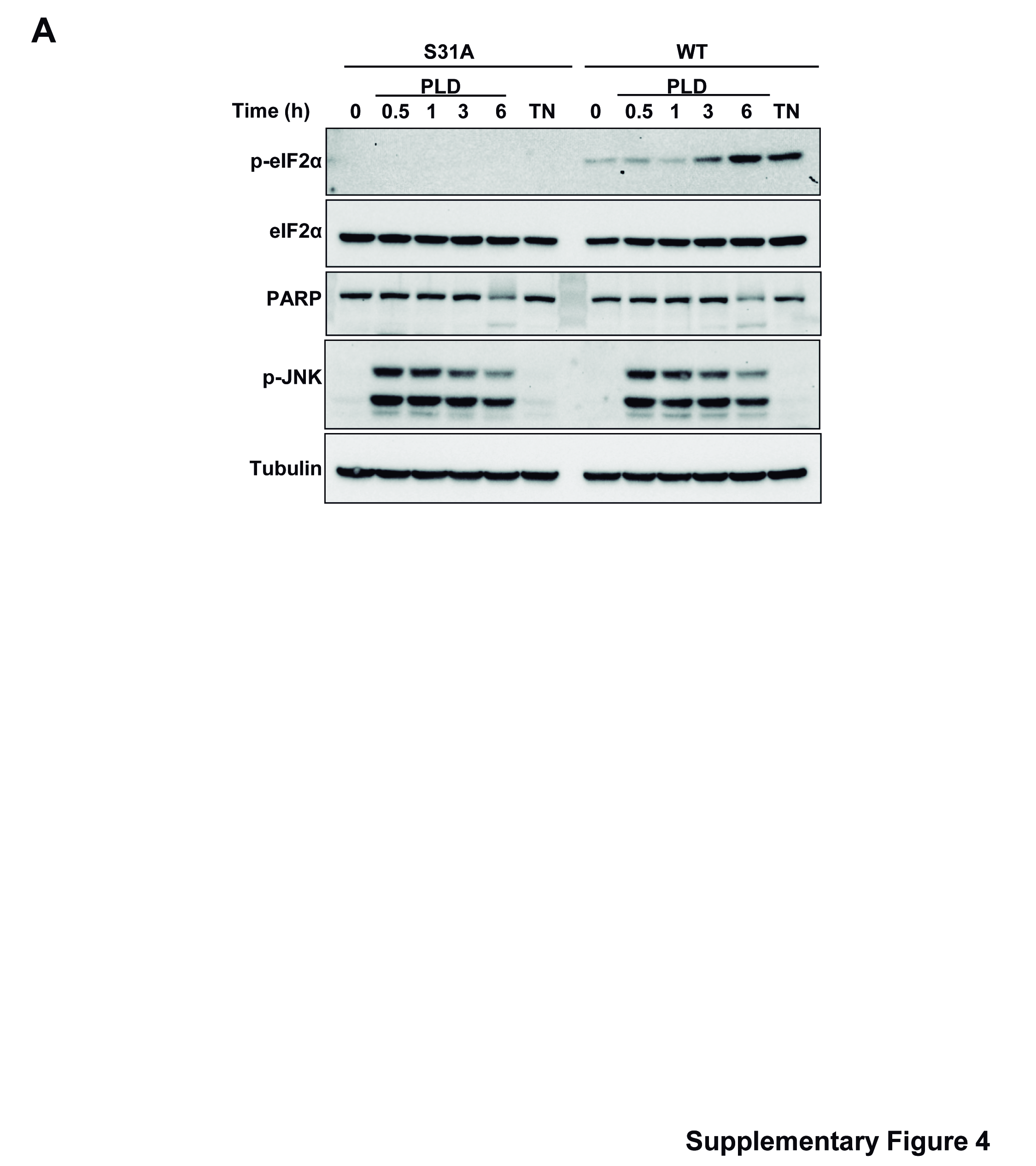

Supplement: Supplementary file 4 — Supplementary Figure 4 [file 41416_2018_336_MOESM4_ESM.tif]
