# Supplementary material for: Binding of eEF1A2 to the RNA-dependent protein kinase PKR modulates its activity and promotes tumour cell survival
Source: Br J Cancer. 2018 Nov 13;119(11):1410–20. doi: 10.1038/s41416-018-0336-y (PMC6265344; doi:10.1038/s41416-018-0336-y)
Supplement: Supplementary file 5 — Supplementary Figure 5 [file 41416_2018_336_MOESM5_ESM.docx]

**SUPPLEMENTARY INFORMATION**

**LEGENDS TO SUPPLEMENTARY FIGURES**

**Supplementary Figure 1: Interaction of eEF1A2 with peroxiredoxin-1 (PRDX) in tumour cells.**

PRDX1, an enzyme involved in the elimination of cellular peroxides, has been shown to interact with eEF1A2, the complex having enhanced anti-oxidative activity^8^. Therefore, it is plausible to infer a direct correlation between the dissociation of eEF1A2:PRDX1 complexes and the increased oxidative stress observed in plitidepsin-treated cells. HeLa cells (WT) or a derivative (PRDX1/A2) overexpressing eEF1A2-GFP and PRDX1-Myc were either left untreated (c) or exposed to 450 nM plitidepsin (PLD) for the indicated times (min.). Whole cell extracts were subjected to immunoprecipitation using anti-GFP (A) or anti-Myc (B) antibodies. Immunoprecipitates were subjected to SDS-PAGE and immunoblotted using antibodies against Myc and eEF1A2, respectively. IP marks protein extract subjected to immunoprecipitation, while INPUT corresponds to 10% of IP. Levels of eEF1A2/PRDX1 complex were quantitated by densitometry. Percentages of precipitated eEF1A2 and PRDX1 from several experiments, with the corresponding standard errors, are plotted in a bar graph beside each Western blot. Differences were considered significant at *P<0.05, **P<0.01, ***P<0.001.

Supplementary Figure 2. Interaction of eEF1A2 with sphingosine kinase (SPHK) in tumour cells.

Sphingosine 1-phosphate (S1P) is a lipid messenger that regulates important cellular processes such as survival, differentiation and migration, and plays an important role in carcinogenesis. S1P is generated through phosphorylation of sphingosine by two sphingosine kinase isoenzymes, namely SPHK1 and SPHK2. Here we show that, plitidepsin does not clearly modify the interaction between eEF1A2 and SPHK. (**A**) HeLa cells (WT) or variants (A2-GFP) overexpressing eEF1A2-GFP were either left untreated (c) or exposed to 450 nM PLD for the indicated times (in minutes). Whole cell extracts were subjected to immunoprecipitation using anti-SPHK1 (upper panel) or anti-SPHK2 (lower panel) antibodies. Immunoprecipitates were subjected to SDS-PAGE and immunoblotted using antibodies against Myc and either SPHK1 or SPHK2. IP marks protein extract subjected to immunoprecipitation, while INPUT corresponds to 10% of IP. (**B**) HeLa WT and APL-R cells were lysed and the SPHK activity determined. Lower panel, Western blot used to measure the level of eEF1A2 protein was determined by immunoblotting with the appropriate antibody. The data are the means + S.E. of three independent experiments each performed in triplicate. Differences were considered significant at *P<0.05, **P<0.01, ***P<0.001. (**C**) Wild-type HeLa cells or a variant cell line overexpressing eEF1A2-Flag were lysed and the SPHK activity determined as described in the text. Lower panel, Western blot performed to measure the level of eEF1A2-Flag protein. SPHK1 activity is expressed as % of the control activity. The data are the means + S.E. of three independent experiments each performed in triplicate. Differences were considered significant at *P<0.05, **P<0.01, ***P<0.001.

**Supplementary Figure 3. Plitidepsin effect on the modulation of the equilibrium sphingosine-1P:ceramide.**

Plitidepsin inhibits both SPHK activity and S1P production through its binding to eEF1A2. (A) Wild-type HeLa cells were treated with vehicle (c), 450 nM PLD, TPA (SPHK activator) or PF-543 (SPHK inhibitor) for the indicated times. Cells were then lysed and the SPHK activity determined (expressed as % of the control). Differences were considered significant at *P<0.05, **P<0.01, ***P<0.001. (B) Wild-type HeLa cells were treated with vehicle (c) or 450 nM PLD for the indicated times and then lysed and extracts analysed for their S-1P levels with an ELISA system as described in Materials and Methods (results expressed as % of the control). Differences were considered significant at *P<0.05, **P<0.01, ***P<0.001. (C) Wild-type HeLa (white bars) and APL-R HeLa (grey bars) cells were treated with 50 nM PLD for the indicated times and total ceramide levels were quantitated and referred to total lipid phosphorus. Differences were considered significant at *P<0.05, **P<0.01, ***P<0.001.

**Supplementary Figure 4. Apoptosis induced by plitidepsin is independent on the phosphorylation status of eIF2α.**

MEF cells expressing wildtype eIF2α (wt) or the S51A mutant version of eIF2α were treated with 450 nM PLD for the indicated times. PARP cleavage and phosphorylated forms of JNK and eIF2α were analysed by Western blotting. Membranes were re-probed with antibodies against total eIF2α and tubulin. Tunicamycin was used as a positive control for eIF2α phosphorylation.
